# Polymorphisms in the Mitochondrial DNA Control Region and Frailty in Older Adults

**DOI:** 10.1371/journal.pone.0011069

**Published:** 2010-06-10

**Authors:** Ann Z. Moore, Mary L. Biggs, Amy Matteini, Ashley O'Connor, Sarah McGuire, Brock A. Beamer, M. Danielle Fallin, Linda P. Fried, Jeremy Walston, Aravinda Chakravarti, Dan E. Arking

**Affiliations:** 1 Department of Epidemiology, Johns Hopkins Bloomberg School of Public Health, Baltimore, Maryland, United States of America; 2 Department of Biostatistics, School of Public Health, University of Washington, Seattle, Washington, United States of America; 3 McKusick-Nathans Institute of Genetic Medicine, Johns Hopkins University School of Medicine, Baltimore, Maryland, United States of America; 4 Division of Gerontology, University of Maryland School of Medicine, Baltimore, Maryland, United States of America; 5 Columbia University Mailman School of Public Health, New York, New York, United States of America; 6 Division of Geriatric Medicine and Gerontology, Department of Medicine, Johns Hopkins University School of Medicine, Baltimore, Maryland, United States of America; Stanford University School of Medicine, United States of America

## Abstract

**Background:**

Mitochondria contribute to the dynamics of cellular metabolism, the production of reactive oxygen species, and apoptotic pathways. Consequently, mitochondrial function has been hypothesized to influence functional decline and vulnerability to disease in later life. Mitochondrial genetic variation may contribute to altered susceptibility to the frailty syndrome in older adults.

**Methodology/Principal Findings:**

To assess potential mitochondrial genetic contributions to the likelihood of frailty, mitochondrial DNA (mtDNA) variation was compared in frail and non-frail older adults. Associations of selected SNPs with a muscle strength phenotype were also explored. Participants were selected from the Cardiovascular Health Study (CHS), a population-based observational study (1989–1990, 1992–1993). At baseline, frailty was identified as the presence of three or more of five indicators (weakness, slowness, shrinking, low physical activity, and exhaustion). mtDNA variation was assessed in a pilot study, including 315 individuals selected as extremes of the frailty phenotype, using an oligonucleotide sequencing microarray based on the Revised Cambridge Reference Sequence. Three mtDNA SNPs were statistically significantly associated with frailty across all pilot participants or in sex-stratified comparisons: mt146, mt204, and mt228. In addition to pilot participants, 4,459 additional men and women with frailty classifications, and an overlapping subset of 4,453 individuals with grip strength measurements, were included in the study population genotyped at mt204 and mt228. In the study population, the mt204 C allele was associated with greater likelihood of frailty (adjusted odds ratio = 2.04, 95% CI = 1.07–3.60, p = 0.020) and lower grip strength (adjusted coefficient = −2.04, 95% CI = −3.33– −0.74, p = 0.002).

**Conclusions:**

This study supports a role for mitochondrial genetic variation in the frailty syndrome and later life muscle strength, demonstrating the importance of the mitochondrial genome in complex geriatric phenotypes.

## Introduction

Mitochondria are a principle contributor to cellular metabolism and produce much of the chemical energy required by cells [1]–[3]. Mitochondria are also integral to some apoptotic processes and are a major production site of reactive oxygen species (ROS) which have both essential and deleterious functions within cells [3]–[7]. Mitochondrial capacity for ATP production and the efficiency of this production are determinants of cellular metabolism, and are thus integral to the dynamics of energy supply and influence metabolic rate of the body on whole [8]. Mitochondria are potential contributors to reductions in the ability to modify resting metabolic rate in response to stressors, and it has been hypothesized that such losses of metabolic plasticity may lead to dysregulation of homeostatic balance and subsequent declines in health [9]. Due to these diverse functions, age-related changes in mitochondria as well as variation in the mitochondrial DNA (mtDNA) have been hypothesized to play a role in degenerative and senescent processes and ultimately contribute to increases in late life vulnerability to chronic disease states, and functional decline. Mitochondrial variation is also hypothesized to influence frailty, a syndrome of decreased reserve and resilience characterized by multi-systemic declines [10], [11]. Indeed, variants in mtDNA have been associated with an increased risk for many chronic diseases and conditions common in older adults including aspects of cardiovascular disease, diabetes, dementias, hearing loss, and cancers [3], [12]–[16].

We hypothesized that the accumulation of environmental exposures across the life course may alter the magnitude of the effect of mtDNA variants on mitochondrial pathways or that inherited patterns of general inefficiency may manifest as observable deficits in later life when accumulated damage is no longer compensated for by other systems increasing susceptibility to the frailty syndrome. In addition, given that the impact of mitochondrial dysfunction may be more pronounced in metabolically active skeletal muscle tissue we further explored a possible intermediate phenotype, grip strength, in order to gain insight into the potential paths linking mtDNA to the frailty phenotype.

## Methods

### Ethics Statement

CHS protocols were approved by each field center's institutional review board; all participants provided written informed consent.

### Study Design

The study population and a small pilot group were selected from the Cardiovascular Health Study (CHS). CHS is a population-based prospective cohort study of the causes of cardiovascular disease in community dwelling adults aged 65 and older; CHS study design, population selection, and protocols have been previously described [17]. Briefly, potential study participants were recruited based upon Medicare eligibility data from four areas: Forsyth County, NC, Sacramento County, CA, Washington County, MD, and Pittsburgh, PA. 5,201 participants were initially enrolled; to improve cohort diversity an additional 687 African-Americans were later recruited. At baseline, June 1989 – June 1990 and November 1992 – June 1993, CHS participants completed an interview and examination that ascertained detailed demographic, health history, and health behavior information as well as clinical and laboratory measurements [17]. Demographic information and anthropometric measurements used for our analyses, including age, sex, race, and BMI, were obtained from CHS baseline interview and examination data. Adjudicated diagnosis of coronary heart disease (CHD), congestive heart failure (CHF), and diabetes mellitus were also drawn from the CHS baseline evaluation [17]. Frail individuals were identified by the presence of three or more of the following previously validated criteria: shrinking, slow walking speed, weakness, self-reported exhaustion, and low physical activity [10], [11]. Isometric grip strength was measured using a Jamar hand-held dynamometer: the strength measure represents the mean of three attempts with the dominant hand [10]. Weakness was defined as a strength measure below the 20th percentile of sex and BMI stratified strength distributions [10].

The pilot study of 154 frail (mean age = 75.00, s.d. = 4.45) and 161 older robust (mean age = 81.35 s.d. = 3.16) individuals was selected from CHS participants who provided consent for genetic studies. In an attempt to maximize the genetic difference between the two groups the pilot participants were selected from the youngest frail and oldest robust participants. We further restricted selection to self-identified whites, due to the limited number of frail blacks in CHS, and matched on sex, resulting in ∼70% females in the pilot group. Individuals with Parkinson's disease, as identified by self-report or medication use, were not eligible for the pilot study, individuals who used Alzheimer's disease related medications, anti-depressants, had a Mini-Mental State Exam score less than 18, BMI greater than 40, or adjudicated history of stroke were also ineligible. “Robust” individuals in the pilot group were the oldest eligible non-frail individuals. All individuals in the CHS study who provided consent for genetic studies were eligible for the full study population. From the 5,077 additional CHS participants with available DNA, but not included in the pilot study, 31 individuals reporting a race/ethnicity other than white or black were excluded. An additional 515 individuals were excluded for indicators of disease (as above) and 96 were excluded for BMI>40. The frailty status of 7 remaining participants was not classified. The final combined population for frailty analyses included 4,459 participants not genotyped in the pilot group plus the 315 pilot participants. Exploratory grip strength analyses excluded individuals based upon the same disease-related criteria but included some individuals that lacked frailty status classification. Participants were excluded that were missing BMI data or had grip strength recorded as <0 kg or >80 kg (n = 317). The final grip strength analyses included 4,453 participants.

### Genotyping

In the pilot study, variants in mtDNA obtained from peripheral lymphocytes were characterized using an oligonucleotide sequencing microarray with probes based upon the Revised Cambridge Reference Sequence (rCRS [NC_012920]): the Human Mitochondrial Resequencing Array 2.0 (Affymetrix, Santa Clara, CA) [18]. Grid-alignment and genotype calls were assigned using RA Tools Ver1.0.5 (http://www.dpgp.org/RA/ra.htm) based on the ABACUS algorithm under a model that assumes a completely haploid genome (i.e. no appreciable levels of heteroplasmy) [19]. An average call rate of 98.6% (range 97.2% to 98.9%) was observed. SNPs were detected at 899 sites, and 209 SNPs with minor allele frequencies (MAF) greater than 1% were included in this analysis.

Individual SNPs selected for follow-up, after demonstrating nominal statistical significance (p<0.05) in pilot analyses, were genotyped with TaqMan assays (Applied Biosystems, Foster City, CA) in all 5,392 CHS participants with genetic consent. Raw data were analyzed using a model that may detect potential heteroplasmy. Genotyping at mt204 was 89.1% complete, and mt228 was 92.0% complete. Frequency of potential heteroplasmy among called genotypes was .003 and <.001 respectively. The concordance between resequencing array and confirmatory TaqMan assay calls was 99.43%. In analyses of mt204 and mt228 using the full population, resequencing array and TaqMan genotypes were combined to minimize missing genotypes. Pilot participants with inconsistent calls across methods were assigned ‘no call’ at the SNP, which resulted in 2 missing genotypes at mt204 and 1 at mt228. Participants with calls indicating potential heteroplasmy were also assigned ‘no call’.

### Statistical Analysis

Student's t-tests with unequal variance and Fisher's exact χ^2^ tests were used to describe the distribution of non-genetic variables of interest across frail and robust individuals among pilot participants and across allele groups in the study population. In the pilot study Fisher's exact χ^2^ tests were used to detect whether the proportion of frail and robust individuals who possessed the minor allele at each of the 209 mtSNPs with MAF >0.01 differed from expectation. The χ^2^ tests were calculated across all participants genotyped at a SNP as well as in sex-stratified groups. In the study population, cross-sectional frailty analyses were stratified by race and sex. Fisher's exact χ^2^ tests were used to compare the proportion of individuals with each allele at selected SNPs. Logistic regression models were used to compare the odds of frailty among allele groups adjusted for age, sex, and/or race. Exploratory cross-sectional grip strength analyses included comparison of the strength measurement across alleles using t-tests with unequal variance stratified as above. The relationship between grip strength and allele was modeled using linear regression. Models were adjusted for age, (age−70)^+^, BMI, BMI^2^, race and/or sex. Grip strength comparisons were repeated after excluding frail participants. Sensitivity analyses compared measures of association when individuals with potential heteroplasmy were included in different allele groups. All analyses were performed using R, version 2.8.1 (R Foundation for Statistical Computing, Vienna, Austria).

## Results

### Association of mtDNA variation with frailty

To assess the potential for a mitochondrial genetic contribution to frailty, common variants in mtDNA, identified using oligonucleotide resquencing arrays, were compared among frail and non-frail individuals in a pilot study. This 315 person group was selected from CHS based upon demographic and disease-related characteristics, focusing on the youngest frail and oldest robust individuals, in an attempt to maximize the genetic difference between the two groups. The pilot group was further restricted to self-identified whites and matched on the proportion of women in the frail and non-frail group. As expected based upon the pilot study selection criteria, the mean age of individuals in the non-frail group (81.35 yrs, s.d. = 3.16) was substantially greater than that of the frail group (75.00 yrs, s.d. = 4.45). Further, the frail participants demonstrated a larger chronic disease burden than non-frail pilot participants with approximately two to four times greater prevalence of diabetes mellitus, coronary heart disease, and congestive heart failure (Table 1).

**Table 1:** Distribution of selected characteristics among all CHS white and black participants with frailty classification (n = 5275) as well as in the pilot population across frailty status.

Characteristic	White			Pilot			Black		
	Non-frail	Frail	p*	Non-frail	Frail	p	Non-frail	Frail	p
	(n = 4223)	(n = 262)		(n = 161)	(n = 154)		(n = 688)	(n = 102)	
Age, mean ± SD	**72.43±5.37**	**77.36±6.36**	**<.001**	**81.35±3.16**	**75.00±4.45**	**<.001**	**72.05±5.09**	**75.44±6.76**	**<.001**
Female, n (%)	**2377 (56.3)**	**174 (66.4)**	**.001**	111 (68.9)	112 (72.7)	.536	**425 (61.8)**	**76 (74.5)**	**.015**
Body mass index, mean ± SD†	26.32±4.33	26.52±5.61	.570	**24.98±3.65**	**26.28±4.75**	**.007**	28.61±5.25	29.00±7.01	.595
Diabetes mellitus, n (%)‡	**571 (13.6)**	**60 (23.1)**	**<.001**	**13 (8.1)**	**38 (24.8)**	**<.001**	161 (24.2)	29 (30.2)	.209
Coronary heart disease, n (%)	**776 (18.4)**	**83 (31.7)**	**<.001**	**28 (17.4)**	**52 (33.8)**	**.001**	127 (18.5)	24 (23.5)	.226
Congestive heart failure, n (%)	**138 (3.3)**	**37 (14.1)**	**<.001**	**6 (3.7)**	**26 (16.9)**	**<.001**	**29 (4.2)**	**14 (13.7)**	**<.001**

*t-test with unequal variance for comparison of continuous characteristics across pilot population groups, Fisher's exact χ^2^ for categorical comparisons.

†missing BMI for some participants: white n_non-frail_ = 4212, n_frail_ = 262; black n_non-frail_ = 686, n_frail_ = 101.

‡missing diabetes mellitus status for some participants: white n_non-frail_ = 4199, n_frail_ = 260; pilot n_non-frail_ = 160, n_frail_ = 153, black n_non-frail_ = 665, n_frail_ = 96.

When comparing SNP differences between frail and non-frail groups in the pilot study, the most statistically significant p-value achieved, 0.006, was observed at a SNP in the D-loop, mt204 (Table S1). A disproportionate number of frail individuals (7/141, 5%) possessed the minor allele (C) at this position compared to the non-frail individuals (0/151). In sex-stratified comparisons the lowest p-values were also observed at SNPs in the D-loop: in men at mt146 (p = 0.003) and in women at mt228 (p = 0.035) (Table S1). The A allele at position 228 was observed in 8/111 (7.2%) frail women but in only 1/110 (0.9%) non-frail women.

The three SNPs that were statistically significant (p<0.05) in the overall or sex-stratified analyses of pilot resequencing array genotypes were selected as candidates for genotyping in the remaining CHS samples; however assay design failed for mt146, which was subsequently dropped from all further analyses. As shown in Table 2, 87 of 3,708 eligible white individuals (2.3%) are carriers of the C allele at mt204. At mt228, The A allele was present in 211 out of 3,821 (5.5%) white individuals (Table S2). The distributions of demographic and disease-related variables were similar across allele groups at mt204 and mt228 in non-pilot participants (Table 2, Table S2).

**Table 2:** Distribution of selected characteristics by mt204 allele in participants stratified by race.

Characteristic				White*						Black		
	All			Pilot			Non-pilot					
	T (n = 3621)	C (n = 87)	p†	T (n = 285)	C (n = 7)	p	T (n = 3327)	C (n = 81)	p	T (n = 540)	C (n = 30)	p
Frail, n (%)	193 (5.3)	8 (9.2)	.142	**134 (47.0)**	**7 (100)**	**.006**	56 (1.7)	2 (2.5)	.648	63 (11.7)	6 (20.0)	.242
Age, mean ± SD	72.77±5.58	72.83±5.78	.922	**78.47±4.90**	**73.0±4.62**	**.020**	72.26±5.35	72.85±5.89	.375	72.54±5.50	71.17±4.28	.101
Female, n (%)	2044 (56.4)	52 (59.8)	.585	203 (71.2)	4 (57.1)	.419	1836 (55.2)	48 (59.3)	.499	331 (61.3)	18 (60.0)	1
Body mass index, mean ± SD ‡	26.14±4.09	26.19±3.97	.913	25.37±4.14	25.55±4.88	.926	26.20±4.07	26.22±3.93	.958	28.04±4.62	29.03±4.83	.284
Diabetes mellitus, n (%)§	503 (13.9)	11 (12.6)	.875	44 (15.5)	1 (14.3)	1	457 (13.8)	9 (11.1)	.623	114 (21.7)	9 (32.1)	.241
Coronary heart disease, n (%)	706 (19.5)	14 (16.1)	.494	71 (24.9)	3 (42.9)	.375	632 (19.0)	11 (13.6)	.252	106 (19.6)	4 (13.3)	.484
Congestive heart failure, n (%)	141 (3.9)	3 (3.4)	1	28 (9.8)	2 (28.6)	.155	113 (3.4)	1 (1.2)	.524	**30 (5.6)**	**5 (16.7)**	**.030**

*The strata labeled “All” includes pilot participants who were not called in array genotyping but were called by the secondary mt204 assay and excludes participants with inconsistent calls across assays.

†t-test with unequal variance for comparison of continuous characteristics, Fisher's exact χ^2^ for categorical comparisons.

‡Missing BMI for some participants: white, all n_T_ = 3613, n_C_ = 87; white, non-pilot n_T_ = 3319, n_C_ = 81; black n_T_ = 538, n_C_ = 30.

§Missing diabetes mellitus status for some participants: white, all n_T_ = 3608, n_C_ = 87; white, pilot n_T_ = 283, n_C_ = 7; white, non-pilot n_T_ = 3316, n_C_ = 81; black n_T_ = 525, n_C_ = 28.

To assess the relationship of variation at mt204 and mt228 with frailty, trends in the frequency of the minor allele at each candidate SNP were observed and logistic regression models were evaluated. While not statistically significant, the direction of the odds ratio for frailty in the group of all CHS participants identifying as white was consistent with pilot data for mt204: in a logistic regression model adjusted for age and sex the odds of frailty were 1.75 (95% CI = 0.74–3.61, p = 0.164) times greater among the group with the C allele than those with the T allele. The non-pilot white participant group includes few frail individuals however adjusted models still indicated a small positive association between the C allele and frailty (Table 3). In the group of participants identifying as black, none of whom were part of the pilot study, a similar trend was observed, odds ratio: 2.33 (95% CI = 0.82–5.74, p = 0.082). Given the consistent directions of association across race/ethnicity stratified groups, combined models assessing mt204 allele were evaluated. The odds of frailty were statistically significantly greater among all participants with the C allele than those with the T allele after adjustment for age, sex, and race, odds ratio: 2.04 (95% CI = 1.07–3.60, p = 0.020). In sex-stratified groupings a stronger association between the minor allele and frailty was observed among men: 3.83 (95% CI = 1.48–8.77, p = 0.003), than among women: 1.33 (95% CI = 0.53–2.86, p = 0.503). Using a model incorporating a sex by allele interaction term, we observed a 2.93-fold (95% CI = 0.87–9.93) increased risk in men relative to women, although this result did not achieve statistical significance (p = 0.079). A consistent association between the mt228 A allele and frailty was not observed in the study population (Table S3). Substantial differences in these associations were not observed in sensitivity analyses including potentially heteroplasmic participants.

**Table 3:** Odds ratios estimating the association of frailty with the C allele at mt204 in the CHS populations observed in stratified multivariate logistic regression models adjusted for age, sex and/or race.

Group			Odds ratio (95% confidence interval) p
Race/ethnicity strata	White	All (n = 3708)	1.75 (0.74, 3.61) .164
		Non-pilot (n = 3408)	1.11 (0.15, 4.70) .904
	Black (n = 570)		2.33 (0.82, 5.74) .082
Combined (n = 4278)			**2.04 (1.07, 3.60) .020**
Sex strata	Female (n = 2445)		1.33 (0.53, 2.86) .503
	Male (n = 1833)		**3.83 (1.48, 8.77) .003**

### Association of mt204 with grip strength

Weakness is a component of the frailty syndrome that may relate closely to mitochondrial variation because it is partially determined by characteristics of skeletal muscle, a metabolically active tissue. Associations of mt204 allele with grip strength were assessed using multiple linear regression models. Lower mean grip strength was observed in groups with the mt204 C allele, consistent with the effect of the C allele on frailty. In multivariate linear regression models adjusting for age, (age−70)^+^, sex, BMI and BMI^2^, the C allele was associated with an approximately two kilogram mean reduction in strength: −2.04 (95% CI = −3.33, −0.74, p = 0.002) (Table 4). In sex-stratified models, the C allele was associated with a greater reduction in strength among men: −3.48 kg (95% CI = −5.94 – −1.02, p = 0.006), than among women: −1.13 kg (95% CI = −2.50–0.24, p = 0.105) (p for interaction = 0.075).

**Table 4:** Mean difference in grip strength (kg) associated with the mt204 C allele estimated by stratified multivariate regression models adjusted for age, (age−70)^+^, BMI, BMI^2^, sex, and race.

Group		Model 1*	Model 2†
		Coefficient (95% confidence interval) p	
Race/ethnicity strata	White (n = 3435)	**−1.60 (−3.10, −0.11) .035**	**−1.63 (−3.12, −0.14) .032**
	Black (n = 558)	**−3.11 (−5.85, −0.37) .027**	**−3.26 (−5.99, −0.54) .019**
Combined (n = 3993)		**−1.80 (−3.10, −0.50) .006**	**−2.04 (−3.33, −0.74) .002**
Sex strata	Female (n = 2274)	−0.87 (−2.26, 0.51) .217	−1.13 (−2.50, 0.24) .105
	Male (n = 1719)	**−3.31 (−5.77, −0.85) .008**	**−3.48 (−5.94, −1.02) .006**

*Adjusted for age, (age−70)^+^, and sex.

†Adjusted for age, (age−70)^+^, BMI, BMI^2^, male sex and/or black race.

To test whether the association with grip strength was driven by the association with frailty, we assessed grip strength after excluding frail participants. The inverse relationship between the C allele and grip strength persisted after excluding frail participants, indicating that the mt204 association with grip strength was not solely a product of its association with frailty (Table S4). To test the reverse hypothesis, that the association with grip strength may be driving the association with frailty, grip strength was introduced into logistic regression models for frailty. The mt204 odds ratio in most models was attenuated but remained positive (Table S5). Further, while the mt204 C allele was not statistically significantly associated with four other phenotypes that help define the frailty syndrome, marginal positive trends were observed for three of the criteria (Table S6).

## Discussion

Given the role of mitochondria in decreased metabolism and increased generation of ROS with aging, there is a strong *a priori* argument that mtDNA variation may contribute to global losses in metabolic plasticity leading to the decreased resilience that is a hallmark of the frailty phenotype. In the present study, analyses of genotypes representing the entire mtDNA obtained through evaluation of a resequencing array under a haploid model in a pilot study of frail and robust individuals generated several promising SNPs. Through follow up genotyping of selected candidates in the entire CHS cohort, we identified mt204 to be associated with frailty. Though less pronounced than in the pilot study, consistently positive associations between the C allele and frailty were observed across all study population groups including participants identifying as black, a group that does not overlap the pilot sample. The influence of mt204 was most apparent after stratification of the population by sex: the odds of frailty among men with the C allele were three times those with the T allele. Further, the absence of variation in the distribution of diabetes mellitus, coronary heart disease, and congestive heart failure by mt204 allele in the full study population suggests that the observed associations with frailty may not be a consequence of an association with chronic disease. Subsequently, an association between mt204 and grip strength was explored, not only because of the contribution of grip strength to frailty assessment, but also because of the dependence of skeletal muscle on metabolic processes. Again, the observed association was more pronounced in males, but was consistent across the entire CHS population and remained even after excluding frail individuals to minimize the potential for bias due to initial selection methods. The presence of an association in the absence of frail participants strongly suggests that the mt204 association with grip strength is not a consequence of its association with frailty. Models for frailty incorporating grip strength and models for other frailty related variables (unintentional weight loss, exhaustion, and low physical activity level) suggest that general skeletal muscle strength, captured here by grip strength, may influence other components of the syndrome, or mt204 may broadly contribute to frailty in a manner that is most apparent in the weakness component.

While mt204 is not in a protein coding region, its location suggests plausible mechanisms for influencing frailty and grip strength. mt204 is within hypervariable segment 2 and has been previously documented as a variant in phylogenetic analyses of the mitochondrial DNA sequence, but is not unique to a single haplogroup [20], [21]. The SNP is also located within the D-loop of the mtDNA control region [20]. mtDNA control region SNPs have been both positively and negatively associated with longevity [22] and mt16189, also a control region variant, has been associated with endometrial cancer and diabetes risk [23], [24]. mt204 is located within the heavy strand origin of replication [25], and thus variation at this SNP may effect the efficiency of mtDNA replication; autosomal genes have been previously linked to mtDNA replication disorders that include muscle myopathy phenotypes [26]. Other SNPs identified in the pilot population, mt146 and mt228, are also located in this region.

While pathways involving variation at mt204 are plausible, it is also possible that the observed associations are due to chance or an unrecognized consequence of population selection criteria. Validation of the mt204 findings in different populations as well as exploration of intermediate phenotypes is necessary. Additionally, several limitations of the present study should be noted. The small size of the pilot study limits statistical power as well as the ability to characterize the effects of less common genetic variants: it is possible that selected candidates do not represent the only mtDNA variants with a potential influence on frailty. In this study we have taken an approach focusing on inherited variation (as measured in DNA isolated from pooled lymphocytes) as opposed to somatic changes, and we are not able to assess the variants of interest in additional tissue types. In our exploration of grip strength we were unable to fully characterize other influences on muscle strength such as muscle mass and neuromuscular control. Finally, the majority of mitochondrial functional and structural genes are located within the autosomal genome: describing variation in these genes may be critical to fully characterizing mitochondrial contributions to frailty. Despite these limitations, we observed statistically significant association between variation in the mitochondrial genome and frailty. Variation in mitochondrial function may lead to homeostatic dysregulation that may be central to this complex geriatric phenotype. The results of this investigation support a role for mitochondrial genetic variation in the frailty syndrome and later life muscle strength.

## Supporting Information

Table S1 (0.04 MB DOC)

Table S2 (0.04 MB DOC)

Table S3 (0.03 MB DOC)

Table S4 (0.03 MB DOC)

Table S5 (0.03 MB DOC)

Table S6 (0.03 MB DOC)
