# Supplementary material for: Polymorphisms in the Mitochondrial DNA Control Region and Frailty in Older Adults
Source: PLoS One. 2010 Jun 10;5(6):e11069. doi: 10.1371/journal.pone.0011069 (PMC2883558; doi:10.1371/journal.pone.0011069)
Supplement: Table S1 — (0.04 MB DOC) [file pone.0011069.s001.doc]

Supplementary Table 1. Among SNPs with MAF>0.01, SNPs corresponding to the 5 most extreme p-values in Fisher’s exact χ^2^ tests comparing the proportion of alleles among frail and non-frail groups in the pilot population and sex-stratified groups.

| Pilot population | | | | | Women | | | | | Men | | | | |
| --- | --- | --- | --- | --- | --- | --- | --- | --- | --- | --- | --- | --- | --- | --- |
| rCRS | Gene | n | MAF | p | rCRS | Gene | n | MAF | p | rCRS | Gene | n | MAF | p |
| **204** | **CR/D** | **292** | **0.024** | **0.0057** | 204 | CR/D | 207 | 0.019 | 0.0595 | **146** | **CR/D** | **92** | **0.076** | **0.0031** |
| 750 | 12S | 314 | 0.013 | 0.0567 | **228** | **CR/D** | **221** | **0.041** | **0.0353** | 204 | CR/D | 85 | 0.035 | 0.0854 |
| 5999 | COI | 315 | 0.013 | 0.0560 | 9380 | COIII | 223 | 0.018 | 0.0597 | 4216 | ND1 | 92 | 0.239 | 0.0538 |
| 9380 | COIII | 315 | 0.016 | 0.0608 | 9716 | COIII | 223 | 0.018 | 0.0597 | 11251 | ND4 | 91 | 0.242 | 0.0512 |
| 10238 | ND3 | 315 | 0.032 | 0.0564 | 13928 | ND5 | 223 | 0.018 | 0.0597 | 15452 | Cytb | 90 | 0.244 | 0.0845 |
| 12501 | ND5 | 315 | 0.032 | 0.0564 | 16162 | CR/D | 223 | 0.031 | 0.0654 | 16126 | CR/D | 92 | 0.239 | 0.0538 |
|  |  |  |  |  | 16526 | CR/D | 222 | 0.032 | 0.0643 |  |  |  |  |  |

CR/D – Control region including Displacement loop; 12S – 12S ribosomal RNA; ND1 – NADH Dehydrogenase subunit 1; COI – Cytochrome c oxidase subunit I; ATPase8 – ATP synthase F0 subunit 8; COIII – Cytochrome c oxidase subunit III; ND3 – NADH dehydrogenase subunit 3; ND4 – NADH dehydrogenase subunit 4; ND5 – NADH dehydrogenase subunit 5; Cytb – Cytochrome b
