# Supplementary material for: Polymorphisms in the Mitochondrial DNA Control Region and Frailty in Older Adults
Source: PLoS One. 2010 Jun 10;5(6):e11069. doi: 10.1371/journal.pone.0011069 (PMC2883558; doi:10.1371/journal.pone.0011069)
Supplement: Table S2 — (0.04 MB DOC) [file pone.0011069.s002.doc]

Supplementary Table 2. Distribution of selected characteristics by mt228 allele in participants stratified by race.

| Characteristic |  | | | White* | | |  | | | Black | | |
| --- | --- | --- | --- | --- | --- | --- | --- | --- | --- | --- | --- | --- |
|  | All | | | Pilot | | | Non-pilot | | |  | | |
|  | G (n = 3610) | A (n = 211) | p† | G (n = 296) | A (n = 16) | p | G (n = 3315) | A (n =194) | p | G (n = 596) | A (n = 9) | p |
| Frail, n (%) | 200 (5.5) | 11 (5.2) | 1 | 142 (48.0) | 10 (62.5) | .310 | 58 (1.7) | 1 (0.5) | .258 | 71 (11.9) | 2 (22.2) | .297 |
| Age, mean ± SD | 72.78 ± 5.57 | 72.45 ± 5.46 | .393 | 78.36 ± 4.87 | 76.25 ± 6.99 | .251 | 72.28 ± 5.35 | 72.10 ± 5.21 | .635 | 72.47 ± 5.38 | 74.11 ± 8.48 | .578 |
| Female, n (%) | 2065 (57.2) | 108 (51.2) | .100 | 212 (71.6) | 9 (56.2) | .256 | 1853 (55.9) | 98 (50.5) | .158 | 362 (60.7) | 5 (55.6) | .744 |
| Body mass index, mean ± SD ‡ | 26.18 ± 4.09 | 26.03 ± 3.89 | .584 | 25.65 ± 4.24 | 25.21 ± 5.28 | .749 | 26.23 ± 4.07 | 26.11 ± 3.76 | .667 | 28.09 ± 4.69 | 28.56 ± 4.33 | .757 |
| Diabetes mellitus, n (%)§ | 497 (13.8) | 32 (15.3) | .537 | 50 (16.9) | 1 (6.7) | .480 | 447 (13.5) | 31 (16.1) | .331 | 133 (23.0) | 4 (44.4) | .224 |
| Coronary heart disease, n (%) | 706 (19.5) | 38 (18.0) | .655 | 73 (24.7) | 4 (25.0) | 1 | 633 (19.1) | 33 (17.0) | .511 | 115 (19.3) | 0 (0) | .219 |
| Congestive heart failure, n (%) | 145 (4.0) | 7 (3.3) | .855 | 30 (10.1) | 2 (12.5) | .673 | 115 (3.5) | 5 (2.6) | .683 | 37 (6.2) | 0 (0) | 1 |

* The strata labeled “All” includes pilot participants who were not called in array genotyping but were called by the secondary mt228 assay and excludes participants with inconsistent calls across assays.

† t-test with unequal variance for comparison of continuous characteristics, Fisher’s exact χ^2^ for categorical comparisons.

‡ Missing BMI for some participants: white, all n_G_ = 3603, n_A_ = 211; white, non-pilot n_G_ = 3308, n_A_ = 194; black n_G_ = 594, n_A_ = 9.

§ Missing diabetes mellitus status for some participants: white, all n_G_ = 3598, n_A_ = 209; white, pilot n_G_ = 295, n_A_ = 15; white, non-pilot n_G_ = 3304, n_A_ = 193; black n_G_ = 579, n_A_ = 9.
