# Supplementary material for: Polymorphisms in the Mitochondrial DNA Control Region and Frailty in Older Adults
Source: PLoS One. 2010 Jun 10;5(6):e11069. doi: 10.1371/journal.pone.0011069 (PMC2883558; doi:10.1371/journal.pone.0011069)
Supplement: Table S3 — (0.03 MB DOC) [file pone.0011069.s003.doc]

Supplementary Table 3. Odds ratios estimating the association of frailty with the A allele at mt228 in the CHS populations observed in stratified multivariate logistic regression models adjusted for age, sex and race.

| Group |  |  | Odds ratio (95% confidence interval) p |
| --- | --- | --- | --- |
| Race/ethnicity strata | White | All (n = 3821) | 1.05 (0.52, 1.90) .892 |
|  |  | Non-pilot (n = 3509) | 0.23 (0.01, 1.25) .171 |
|  | Black (n = 605) |  | 1.72 (0.23, 8.27) .538 |
| Combined (n = 4426) |  |  | 1.08 (0.57, 1.89) .798 |
| Sex strata | Female (n = 2540) |  | 1.52 (0.71, 2.90) .238 |
|  | Male (n = 1886) |  | 0.54 (0.13, 1.53) .311 |
