# Supplementary material for: Polymorphisms in the Mitochondrial DNA Control Region and Frailty in Older Adults
Source: PLoS One. 2010 Jun 10;5(6):e11069. doi: 10.1371/journal.pone.0011069 (PMC2883558; doi:10.1371/journal.pone.0011069)
Supplement: Table S4 — (0.03 MB DOC) [file pone.0011069.s004.doc]

Supplementary Table 4. mt204 C allele coefficients from stratified multivariate regression models estimating association with grip strength adjusted for age, (age-70)^+^, BMI, BMI^2^, sex, and race. Excluding individuals classified as frail.

| Group |  | Model 1* | Model 2† |
| --- | --- | --- | --- |
|  |  | Coefficient (95% confidence interval) p | |
| Race/ethnicity strata | White (n = 3249) | -1.52 (-3.07, 0.03) .055 | **-1.55 (-3.09, -0.002) .05** |
|  | Black (n = 490) | -2.34 (-5.26, 0.59) .118 | -2.51 (-5.41, 0.40) .092 |
| Combined (n = 3739) |  | **-1.47 (-2.83, -0.10) .035** | **-1.74 (-3.10, -0.39) .012** |
| Sex strata | Female (n = 2110) | -0.67 (-2.10, 0.75) .352 | -0.98 (-2.38, 0.42) .170 |
|  | Male (n = 1629) | **-2.87 (-5.52, -0.21) .034** | **-3.06 (-5.71, -0.41) .023** |

* Adjusted for age, (age-70)^+^, and sex.

† Adjusted for age, (age-70)^+^, BMI, BMI^2^, male sex and/or black race.
