# Supplementary material for: Polymorphisms in the Mitochondrial DNA Control Region and Frailty in Older Adults
Source: PLoS One. 2010 Jun 10;5(6):e11069. doi: 10.1371/journal.pone.0011069 (PMC2883558; doi:10.1371/journal.pone.0011069)
Supplement: Table S5 — (0.03 MB DOC) [file pone.0011069.s005.doc]

Supplementary Table 5. Odds ratios estimating the association of frailty with the C allele at mt204 in participants with frailty status and grip strength measurement observed in stratified multivariate logistic regression models.

| Group |  | Model 1* | Model 2† |
| --- | --- | --- | --- |
|  |  | Odds ratio (95% confidence interval) p | |
| Race/ethnicity strata | White (n = 3442) | 2.12 (0.91, 4.37) .058 | 1.85 (0.76, 3.98) 0.14 |
|  | Black (n = 560) | 2.28 (0.81, 5.62) .090 | 1.68 (0.54, 4.51) .331 |
| Combined (n = 4002) |  | **2.28 (1.20, 4.04) .007** | 1.86 (0.94, 3.43) .059 |
| Sex strata | Female (n = 2278) | 1.55 (0.62, 3.33) .299 | 1.56 (0.61, 3.45) .307 |
|  | Male (n = 1724) | **4.02 (1.54, 9.30) .002** | **2.80 (0.98, 7.13) .040** |

* Adjusted for age, sex and/or race.

† Model 1 plus grip strength.
