# Supplementary material for: Polymorphisms in the Mitochondrial DNA Control Region and Frailty in Older Adults
Source: PLoS One. 2010 Jun 10;5(6):e11069. doi: 10.1371/journal.pone.0011069 (PMC2883558; doi:10.1371/journal.pone.0011069)
Supplement: Table S6 — (0.03 MB DOC) [file pone.0011069.s006.doc]

Supplementary Table 6. Odds ratios estimating the association of other frailty related phenotypes with the C allele at mt204 in CHS participants with phenotype and genotype measurements observed in multivariate logistic regression models adjusted for age, sex and race.

| Phenotype | n | Odds ratio (95% confidence interval) p |
| --- | --- | --- |
| Shrinking* | 4063 | 1.16 (0.45, 2.48) .728 |
| Exhaustion† | 4279 | 1.40 (0.88, 2.15) .142 |
| Slowness‡ | 4225 | 1.27 (0.70, 2.18) .405 |
| Low activity§ | 4268 | 1.07 (0.66, 1.67) .766 |

* Loss of more than 10lbs. unrelated to diet or exercise.

† In the last week unable to get going or felt everything done an effort a moderate amount of time or most of the time.

‡ 15ft. timed walk ≥7 seconds for women ≤159cm tall and men ≤173cm, ≥6 seconds for women >159 cm tall and men >173cm.

§ Less than 270 kcal for women or 383 kcal for men on the modified Minnesota Leisure Time Activity Questionnaire or Continuous kcal.
